# Effect of Neuromuscular Electrical Stimulation for Older Critically Ill Patients in the ICU: A Randomized Controlled Trial

**DOI:** 10.1097/CCE.0000000000001345

**Published:** 2025-11-25

**Authors:** Kazuhiro Yokobatake, Hiroaki Kitaoka, Atsushi Morizane, Kensaku Kashima, Daichi Nishimori, Shingo Nishimura, Yumi Sakyo, Shinya Takeuchi, Yasumasa Kawano, Tomoko Sugimura

**Affiliations:** 1 Department of Medical Technology Rehabilitation, Kochi Health Sciences Center, Kochi, Japan.; 2 Department of Cardiology and Geriatric, Kochi Medical School, Kochi, Japan.; 3 Critical Care and Emergency Center, Kochi Health Sciences Center, Kochi, Japan.; 4 Department of Nursing, Kochi Health Sciences Center, Kochi, Japan.; 5 Department of Disaster and Emergency Medicine, Kochi Medical School, Kochi Japan.; 6 Department of Emergency and General Medicine, Fukuoka University Chikushi Hospital, Chikusino, Japan.; 7 Department of Emergency Medicine, Graduate School of Medicine, University of the Ryukyus, Okinawa, Japan.

**Keywords:** critical illness patients, hand-held dynamometer, neuromuscular electrical stimulation, older, quadriceps isometric strength

## Abstract

**OBJECTIVES:**

In the ICU, the optimal patient population for neuromuscular electrical stimulation (NMES) and the most appropriate evaluation tools remain unclear. This study aimed to assess whether combining early mobilization with NMES in older critically ill patients improves lower limb muscle strength and physical function at hospital discharge.

**DESIGN:**

Assessor-blinded, randomized controlled trial.

**SETTING:**

A single-center, emergency and critical care center ICU in Japan.

**PATIENTS:**

Patients 65 years old or older with an Acute Physiology and Chronic Health Evaluation II (APACHE II) score greater than 20 admitted to the ICU.

**INTERVENTIONS:**

The participants were randomly assigned to the NMES group (NMES in addition to early mobilization) or the control group (early mobilization alone).

**MEASUREMENTS AND MAIN RESULTS:**

The primary outcome was quadriceps isometric strength (QIS), which was measured using a hand-held dynamometer to ensure objective assessment. QIS values were normalized to body weight. Outcome assessors were blinded to group allocation. A total of 44 patients were randomized, and 32 completed the study (NMES group: 17; control group: 15). The mean age was 77.6 ± 6.5 years, and the mean APACHE II score was 29.7 ± 6.3. NMES was performed for an average of 9.6 ± 4.8 days. There were no baseline differences between groups. At hospital discharge, the mean QIS was 0.46 ± 0.13 kgf/kg in the NMES group and 0.30 ± 0.13 kgf/kg in the control group (mean difference, 0.16; 95% CI, 0.07–0.25; *p* = 0.002). Secondary outcomes, including the 6-minute walk distance and the Barthel Index, were also greater in the NMES group.

**CONCLUSIONS:**

NMES combined with early mobilization improved lower limb muscle strength and functional outcomes in older ICU patients.

**KEY POINTS**

**Question:** Can neuromuscular electrical stimulation (NMES) help prevent muscle weakness in critically ill older patients, and is there a more objective method for assessing muscle strength?

**Findings:** In critically ill older patients, the addition of NMES to early mobilization significantly attenuated the decline in quadriceps isometric strength, as measured by a hand-held dynamometer at discharge. It also improved 6-minute walk distance and activities of daily living performance.

**Meaning:** For critically ill older patients, NMES added to early mobilization may be an effective therapeutic strategy, and a hand-held dynamometer offers a more objective method for assessing muscle strength.

Muscle weakness in critically ill patients admitted to the ICU is referred to as ICU-acquired weakness (ICU-AW). It is defined as clinically detected weakness in critically ill patients in whom there is no plausible etiology other than critical illness (1). The prevalence of ICU-AW varies depending on illness severity and the diagnostic methods employed but is reported to be around 50% (2). No effective pharmacological prevention of ICU-AW has been established; however, early mobilization tailored to each patient’s condition, together with evidence-based ICU practices such as the ABCDEF bundle, has been shown to effectively mitigate muscle weakness (3, 4). Nonetheless, many critically ill patients are unable to mobilize due to unstable clinical conditions or medical and surgical contraindications to out-of-bed activity. Muscle wasting and functional decline in this population are driven by systemic inflammation, with the most pronounced deterioration occurring within a few hours to a few days after ICU admission (5). Neuromuscular electrical stimulation (NMES) is widely recognized for preserving muscle mass and function in immobilized patients across various conditions (6). A group of intensive care specialists in the Netherlands has recommended the application of NMES during the early phase of rehabilitation, particularly when voluntary muscle contractions are not feasible, as a practical rehabilitation strategy (7). However, a recent meta-analysis reported no significant improvement in muscle strength, as measured by the Medical Research Council sum score (MRCss), following NMES (8, 9). Furthermore, international clinical practice guidelines for the management of critically ill patients have yet to reach a consensus on the use of NMES (10, 11). In the ICU, a diverse population with varying diseases, severity levels, ages, prehospital functional status, comorbidities, and degrees of frailty is treated. Therefore, the need to identify responders to NMES and the limitations of muscle strength assessment using the MRCss have been noted (9, 12–14).

To address these issues, we tested the hypothesis that an intervention combining early mobilization with NMES in older critically ill patients in the ICU would improve lower limb skeletal muscle strength, as measured by a hand-held dynamometer (HHD), and their physical function at hospital discharge.

## MATERIALS AND METHODS

### Study Design and Patient Selection

This study was an assessor-blinded, randomized controlled trial. This study was conducted in accordance with the ethical standards of the institutional review board (IRB) of Kochi Health Sciences Center and the 1975 Declaration of Helsinki. The study protocol was approved by the IRB of our hospital (approval number: 201029; study title: “Effect of neuromuscular electrical stimulation for older critically ill patients in the intensive care unit: a randomized controlled trial”; approved on September 11, 2020) and was prospectively registered with the University Hospital Medical Information Network (UMIN000042154). Informed consent was obtained from patients or surrogate decision-makers before randomization. In all cases, patients were informed about their participation in the study upon regaining consciousness. Patients and members of the public were not involved in the study design; however, patients contributed to the continuation of the NMES and their participation in the study. The reporting of this study adhered to the Consolidated Standards of Reporting Trials reporting guidelines (15).

The study was conducted in a 12-bed ICU at Kochi Health Sciences Center, Japan, with a registration period from October 2020 to April 2024 and a 6-month follow-up ending in October 2024. Patients were screened upon ICU admission and enrolled within 24 hours if they: 1) were 65 years old or older and 2) had an Acute Physiology and Chronic Health Evaluation II (APACHE II) score greater than 20. Exclusion criteria were: 1) an expected ICU stay less than 72 hours; 2) a Clinical Frailty Scale (CFS) greater than or equal to 5 (patients who are not independent in activities of daily living [ADLs] even with the use of walking aids); 3) inability to walk at discharge due to stroke, neuromuscular disease, or trauma; 4) pacemaker implantation; 5) poorly controlled malignancy; 6) a designated “Do Not Attempt Resuscitation” order; 7) severe dementia; 8) COVID-19; 9) suicidal attempt; or 10) inability to obtain consent.

### Randomization and Blinding

An independent statistician, who was not involved in the treatment interventions, generated the random numbers using a computer and created the allocation sequence with block sizes of 2 or 4. The masked allocation sequence was opened sequentially by the intervention team after patient enrollment was confirmed, thereby determining group assignment. Participants were randomly assigned in a 1:1 ratio to receive either NMES in addition to early mobilization (NMES group) or early mobilization alone (control group). Due to the nature of the NMES intervention, blinding of the treating clinicians, rehabilitation staffs and patients was not feasible; however, rehabilitation staffs involved after ICU discharge and outcome assessors remained blinded to group allocation.

### Early Mobilization Program

Our ICU adheres to the ABCDEF bundle (3, 4, 11) and follows protocols based on a Japanese study on the safety of early mobilization (16). A five-step progressive program began with in-bed range-of-motion exercises and advanced through sitting, standing, and walking (**Supplemental Digital Content**, **Study Protocol**, https://links.lww.com/CCX/B577). Early rehabilitation was initiated within 48 hours of ICU admission and delivered by a multidisciplinary team comprising dedicated ICU physical therapists, board-certified intensivists (certified by the Japanese Society of Intensive Care Medicine), nurses, and clinical engineers. The team held daily conferences to review each patient’s status and set mobilization goals. If patients did not meet the discontinuation criteria and demonstrated the ability to walk, gait training was actively initiated. The early mobilization program was implemented 7 days a week, and after ICU discharge, patients received enhanced rehabilitation provided by physical therapists and nurses to actively support the recovery of their ADLs.

### NMES Protocol

In the NMES group, belt-type skeletal muscle electrical stimulation (G-TES; Homer Ion Corp., Tokyo, Japan) was applied in addition to the early mobilization program. Belt electrodes were attached to the patient’s proximal and distal thighs and ankles (Supplemental Digital Content, **Supplementary Picture**, https://links.lww.com/CCX/B577). The NMES settings were a frequency of 20 Hz, a pulse width of 250 μs, and a duty cycle of 5 seconds of stimulation followed by a 2-second pause. The intensity was set to the maximum level that induced visible muscle contraction without causing pain. NMES began within 48 hours of ICU admission and was administered daily for 60 minutes, at a different time from the early mobilization sessions. During treatment, a physical therapist, nurse, and intensivist monitored the patient’s responses, facial expressions, and vital signs. Efforts were made to maximize muscle contraction during NMES. Treatment was continued until the patient was able to walk 100 meters continuously, regardless of the use of walking aids or assistance.

### Outcome Measures

The primary outcome of this study was quadriceps isometric strength (QIS), normalized by body weight. An HHD is widely used in clinical practice to objectively assess QIS and has demonstrated reliability across various patient populations (17–19). In this study, an HHD (µ Tas F-1, ANIMA, Tokyo, Japan) was used for muscle strength assessment. Measurements were taken when sedation was lifted and patients were initially able to get out of bed, as well as at hospital discharge. Each measurement was performed twice on each side while the patient was seated with 90° of knee flexion. QIS was calculated by dividing the highest measured value by body weight (17, 18).

Secondary outcomes included the MRCss and grip strength, both of which were assessed alongside QIS at the initial out-of-bed and hospital discharge. Thigh circumference (15 cm above the knee) was recorded at the initial intervention and hospital discharge. Exercise tolerance was evaluated by continuous walking distance at ICU discharge and the 6-minute walk distance (6MWD) (20) at hospital discharge. One-leg standing time was measured at hospital discharge to assess balance. Functional independence was evaluated using the Barthel Index (21) at both ICU discharge and hospital discharge. To assess prognosis and frailty at 6 months, CFS (22) was recorded through outpatient visits or telephone interviews.

### Sample Size Calculation

A power analysis using G*Power 3.1 for Mac (Heinrich Heine University, Düsseldorf, Germany) was conducted during the study planning phase. No previous studies have quantitatively evaluated the effect of NMES on muscle strength in critically ill ICU survivors using QIS. Among community-dwelling individuals over 75 years old, the QIS is approximately 0.40 kgf/kg (23, 24), whereas critically ill ICU survivors have been reported to have a QIS of 0.24 kgf/kg at discharge (25). Assuming a difference of 0.08 kgf/kg in QIS at discharge with a sd of 0.1 kgf/kg, we calculated the required sample size using an alpha of 0.05 and a power of 80%. As a result, 26 patients per group were needed, with a target enrollment of 60 patients to account for mortality and exclusions due to complications.

### Statistical Analysis

Data analysis included all randomized patients, excluding those who died or for whom outcome assessment was not feasible. Baseline demographic and clinical characteristics, including age, sex, body mass index at admission, pre-admission CFS, APACHE II score (26), SOFA score (27), the number of patients with a positive disseminated intravascular coagulation score according to the Japanese Association for Acute Medicine (28), and diagnosis at admission, were summarized using descriptive statistics. Adjuvant therapies in the ICU, such as mechanical ventilation, renal replacement therapy, steroid therapy, analgesics, sedatives, vasoactive drugs, muscle relaxants, and nutritional support, were also evaluated.

The Shapiro-Wilk test was used to assess normality of continuous variables. Between-group comparisons were performed using the unpaired *t* test or Mann-Whitney *U* test for continuous variables, and Fisher exact test for dichotomous variables. Normally distributed continuous variables are presented as the mean ± sd, non-normally distributed variables are presented as the median and interquartile range (IQR), and categorical variables are presented as counts and percentages. Between-group differences in the primary and secondary outcomes are reported as mean differences with 95% CIs and *p* values. For data that were non-normally distributed, median differences were reported, and 95% CI were calculated using the bootstrap method (1000 resampled datasets). Differences for dichotomous variables are presented as absolute risk reduction, along with risk difference and the 95% CI of the odds ratio. All statistical analyses were performed using SPSS Statistics, Version 27.0 (IBM Corp., Armonk, NY). A two-tailed *p* value of less than 0.05 was considered statistically significant in all analyses.

## RESULTS

### Patient and Basic Characteristics

**Figure 1.**
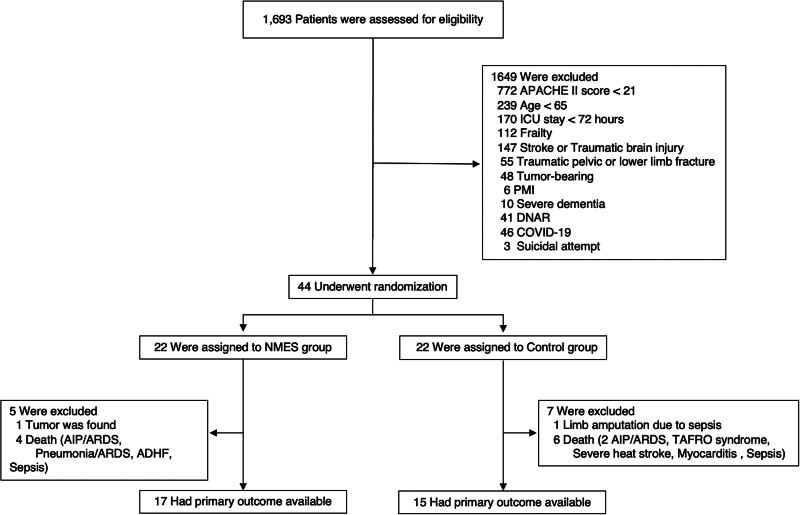
Flowchart of screening and randomization. ADHF = acute decompensated heart failure, AIP = acute interstitial pneumonia, APACHE = Acute Physiology and Chronic Health Evaluation, ARDS = acute respiratory distress syndrome, DNAR = do-not-attempt resuscitation, NMES = neuromuscular electrical stimulation, PMI = pacemaker implantation.

Of the 1693 patients admitted to the ICU and assessed for eligibility, 44 were enrolled and randomized to the NMES group (*n* = 22) or the control group (*n* = 22; **Fig. 1**). The following patients were excluded from the analysis: in the NMES group, one patient who required additional treatment for an incidentally discovered tumor, and four who died after ICU discharge during their general ward stay. In the control group, one patient underwent limb amputation due to sepsis-induced peripheral circulatory failure, two died in the ICU, and four died during the general ward stay after ICU discharge. Thus, primary and secondary outcomes were available for 32 of the 44 patients (72.3%) in the intention-to-treat population (17 in the NMES group and 15 in the control group).

The demographic and clinical characteristics of the patients at baseline are shown in **Table 1**. The study groups had similar baseline characteristics. The mean age was 77.6 ± 6.5 years, and 67% of the patients were male. Premorbid frailty, assessed by the CFS, had a median score of 3 (IQR, 3–3), indicating well-controlled medical conditions and, if present, only mild frailty. The mean APACHE II score was 29.7 ± 6.3 points. Ninety-four percent of patients required mechanical ventilation, and 53% experienced septic shock, as defined by the Sepsis-3 criteria. In the NMES group, the intervention was administered for an average of 9.6 ± 4.8 days, with no adverse events requiring therapeutic intervention. Sufficient muscle contractions were achieved during sedation, and following sedation discontinuation, the stimulation intensity was adjusted as needed to manage NMES-induced pain or discomfort, allowing completion of the intervention in all cases.

**TABLE 1.** Patient Characteristics

Variable	Neuromuscular Electrical Stimulation Group (*n* = 17)	Control Group (*n* = 15)	*p*
Age, mean (sd), yr	77.1 (7.3)	78.1 (5.6)	0.65
Sex (male), *n* (%)	12 (71)	9 (60)	0.71
Body mass index, mean (sd), kg/m^2^	22.4 (2.7)	23.6 (3.9)	0.31
Premorbid Clinical Frailty Scale, median (IQR)	3 (3–4)	3 (3–3)	0.80
Acute Physiology and Chronic Health Evaluation II score, mean (sd)	29.8 (6.7)	29.5 (6.1)	0.90
Sequential Organ Failure Assessment, mean (sd)	11.2 (2.2)	10.8 (3.1)	0.69
Maximum C-reactive protein, mean (sd), mg/dL	20.8 (8.4)	19.3 (9.1)	0.64
ICU admission diagnosis, *n* (%)			0.99
Cardiovascular	8 (47)	7 (47)	
Gastrointestinal	5 (29)	5 (33)	
Respiratory	3 (18)	2 (13)	
Infections	1 (6)	1 (7)	
Sepsis shock, *n* (%)	10 (59)	7 (47)	0.74
Disseminated intravascular coagulation, *n* (%)	6 (35)	6 (40)	0.99
Post-surgery, *n* (%)	7 (41)	9 (60)	0.48
Mechanical ventilation, *n* (%)	16 (94)	14 (93)	0.99
Mechanical ventilation days, median (IQR), d	4 (4–5)	6 (4–7)	0.07
Sedation duration days, median (IQR), d	4 (3–5)	4 (3–6)	0.59
Opioids duration days, median (IQR), d	4 (4–6)	5(4–8)	0.46
Renal replacement therapy, *n* (%)	5 (29)	2 (13)	0.40
Steroids, *n* (%)	9 (53)	4 (27)	0.17
Vasopressor agents, *n* (%)	16 (94)	15 (100)	0.99
Neuromuscular blocking agents, *n* (%)	1 (6)	0 (0)	0.99
Enteral nutrition or total parenteral nutrition start day, median (IQR)	3 (3–4)	3 (3–5)	0.77

IQR = interquartile range.

### Primary Outcome

**Figure 2.**
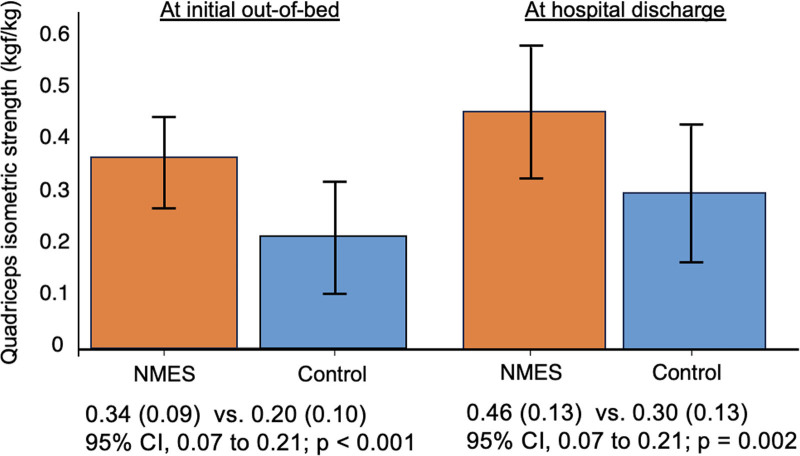
Primary outcome of quadriceps isometric strength (QIS). Mean difference in the QIS between the groups at the initial assessment and at hospital discharge. The mean difference was 0.14 kgf/kg (95% CI, 0.07–0.21 kgf/kg) at the initial assessment and 0.16 kgf/kg (95% CI, 0.07–0.25 kgf/kg) at hospital discharge. NMES = neuromuscular electrical stimulation.

At the initial out-of-bed assessment, QIS was 0.34 ± 0.09 kgf/kg in the NMES group and 0.20 ± 0.10 kgf/kg in the control group (mean difference, 0.14 kgf/kg; 95% CI, 0.07–0.21; *p* < 0.001). At hospital discharge, QIS was 0.46 ± 0.13 kgf/kg in the NMES group and 0.30 ± 0.13 kgf/kg in the control group (mean difference, 0.16 kgf/kg; 95% CI, 0.07–0.25; *p* = 0.002; **Fig. 2**).

### Secondary Outcomes

The results for the secondary outcomes are presented in **Table 2**. The time to initial out-of-bed mobilization did not differ between the two groups, and although the NMES group tended to begin walking earlier, this difference was not statistically significant (median difference, –2 d; 95% CI, –3 to 1; *p* = 0.31). The median MRCss at the initial assessment was higher in the NMES group (38 points; IQR, 36–44 points) than in the control group (36 points; IQR, 34–36 points; median difference, 2 points; 95% CI, 0.1–8 points; *p* = 0.037). At hospital discharge, the median MRCss was 52 points (IQR, 48–55 points) in the NMES group and 51 points (IQR, 45–58 points) in the control group, with no significant difference (median difference, 1 point; 95% CI, –8 to 7 points; *p* = 0.90). The median 6MWD at hospital discharge was 342 meters (IQR, 295–370 meters) in the NMES group and 220 meters (IQR, 147–293 meters) in the control group (median difference, 122 meters; 95% CI, 38–194 meters; *p* = 0.006). Although the Barthel Index did not differ significantly between the groups at ICU discharge (*p* = 0.24), the median score at hospital discharge was 100 points (IQR, 95–100 points) in the NMES group and 75 points (IQR, 58–95 points) in the control group (median difference, 25 points; 95% CI, 0.1–45 points; *p* = 0.008). The median CFS at hospital discharge was 4 (IQR, 3–4) in the NMES group and 6 (IQR, 4–6) in the control group (median difference, –2; 95% CI, –3 to –0.1; *p* = 0.011). Patients who did not regain functional independence and were unable to be discharged home were transferred to hospitals where rehabilitation could be continued.

**TABLE 2.** Mobilization in the ICU and Secondary Outcomes

Variable	NMES Group (*n* = 17)	Control Group (*n* = 15)	Between-Group Difference (95% CI)	*p*
Rehabilitation interventions				
Rehabilitation start day, median (IQR)	2 (2–2)	2 (2–2)	0 (0–0)	0.58
Sitting start day, median (IQR)	5 (4–7)	6 (4–7)	–1 (–2 to 2)	0.50
Initial out of bed start day, median (IQR)	6 (5–7)	6 (5–8)	0 (–2 to 1)	0.43
Walking start day, median (IQR)	6 (5–8)	8 (6–9)	–2 (–3 to 1)	0.31
NMES days, mean (sd), d	9.6 (4.8)	No		
ICU length of stay, median (IQR), d	7 (6–8)	8 (7–11)	–1 (–5 to 1)	0.21
Hospital length of stay, median (IQR), d	23 (20–31)	24 (19–32)	–1 (–10 to 9)	0.82
MRC sum score				
At initial out of bed, median (IQR)	38 (36–44)	36 (34–36)	2 (0.1–8)	0.037
At hospital discharge, median (IQR)	52 (48–55)	51 (45–58)	1 (–8 to 7)	0.90
ICU-acquired weakness, MRC sum score < 48 points				
At initial out of bed, *n* (%)	14 (82.4)	14 (93.3)	–10.9 (0.2–168)	0.60
At hospital discharge, *n* (%)	3 (17.6)	4 (26.7)	–9.1 (0.2–14)	0.68
Thigh circumference				
At rehabilitation start, mean (sd), cm	43.4 (3.5)	42.8 (5.6)	0.6 (–2.8 to 3.9)	0.72
At hospital discharge, mean (sd), cm	39.8 (3.2)	38.7 (5.9)	1.1 (–2.2 to 4.5)	0.50
Hand grip strength				
At initial out of bed, mean (sd), kgf	15.4 (6.5)	11.4 (8.7)	4.0 (–1.5 to 9.6)	0.14
At hospital discharge, mean (sd), kgf	19.7 (6.5)	16.0 (9.9)	3.7 (–2.3 to 9.7)	0.21
Continuous gait distance at ICU discharge, median (IQR), m	25 (0–100)	0 (0–55)	25 (–35 to 100)	0.16
6-min walk distance at hospital discharge, median (IQR), m	342 (295–370)	220 (147–293)	122 (38–194)	0.006
Gait speed at hospital discharge				
Normal walk, mean (sd), m/s	0.81 (0.24)	0.71 (0.25)	0.10 (–0.09 to 0.28)	0.30
Fast walk, mean (sd), m/s	1.11 (0.33)	0.99 (0.30)	0.12 (–0.12 to 0.36)	0.32
Standing on one leg, median (IQR), s	7.9 (4.5–21)	2.9 (0.8–4.9)	5 (0.1–17.6)	0.012
Barthel index score				
At ICU discharge, median (IQR)	15 (5–35)	10 (5–20)	5 (–10 to 25)	0.24
At hospital discharge, median (IQR)	100 (95–100)	75 (58–95)	25 (0.01–45)	0.008
CFS at discharge, median (IQR)	4 (3–4)	6 (4–6)	–2 (–3 to –0.1)	0.011
After hospital discharge, home, *n* (%)	11 (64.7)	5 (33.3)	31.4 (0.70–20)	0.16
CFS after 6 mo, median (IQR)	4 (3–4)	4 (3–6)	0 (–2 to 1)	0.21

CFS = Clinical Frailty Scale, IQR = interquartile range, MRC = Medical Research Council, NMES = neuromuscular electrical stimulation.

## DISCUSSION

In this randomized controlled trial involving older critically ill patients admitted to the ICU, the addition of NMES to early mobilization prevented lower limb muscle weakness at both the initial assessment and hospital discharge, compared with early mobilization alone. As a result, it also contributed to improvements in the 6MWD, Barthel Index, and frailty status. To our knowledge, this is the first interventional trial to demonstrate the effectiveness of NMES in critically ill older ICU patients using muscle strength assessment performed with an HHD.

To date, no specific subgroup of critically ill patients most likely to respond to physical rehabilitation or NMES has been identified (29). The frequency of ICU-AW varies widely across studies due to heterogeneity in disease type and severity, age, baseline health status, and frailty, all of which affect short- and long-term outcomes (9, 12, 30). In addition, the aging population requiring intensive care in developed countries is a growing concern. The severity of critical illness and advanced age are nonmodifiable risk factors for ICU-AW (12, 30). Recent systematic reviews have reported that NMES significantly improves muscle strength in critically ill patients, consistent with our findings (9, 31). However, these reviews did not adjust for patient age or other background factors as potential effect modifiers, and thus, the population for whom NMES is most effective remains uncertain. To address these challenges, we included older adults who were independent in their ADLs before hospitalization, aiming to reduce variability in baseline muscle strength across the study population. Consequently, ICU-AW was observed in 88.5% of patients at their initial out-of-bed assessment. Our findings suggest that older critically ill patients could benefit from NMES.

Another gap in the evidence may be that use of the MRCss for muscle strength assessment influences evaluation of NMES effects. Although the MRCss is recommended for diagnosing ICU-AW (32, 33), it has several limitations, including a ceiling effect, limited sensitivity to mild ICU-AW, difficulty distinguishing between MRC grades 3 and 4, its ordinal nature, and weak correlation with physical function (9, 12–14, 33). In this study, using QIS measured by an HHD as the primary outcome enabled detection of subtle changes in muscle strength not captured by the MRCss at discharge. Previous systematic review (8), which included small samples and older patients, may have failed to detect changes in muscle strength using MRCss. Although QIS assessment using HHD has not yet been widely adopted in ICU patients, its reliability has been established (14, 34). Reduced QIS is an independent predictor of all-cause mortality in apparently healthy adults (35) and is associated with increased mortality risk in patients with coronary artery disease, heart failure, and various acute and chronic conditions (36–38). QIS plays a crucial role in maintaining mobility in older adults (39) and is associated with functional capacity in daily activities, with a reported threshold of 0.27 kgf/kg required for independent ADL performance (40). The NMES group exceeded this threshold from the initial assessment, suggesting a potential impact on clinically meaningful outcomes such as the 6MWD, balance ability, and Barthel Index scores. Quantifying muscle strength in survivors of critical illness may facilitate detection of subtle changes and enable assessment of intervention effects, early mobility, and potential use in prognostic prediction (30). Furthermore, the core outcome set focusing on physical rehabilitation interventions in critically ill adults recommends evaluating physical function, exercise capacity, and ADLs (41). Our study outcomes align with this core outcome set and, although the sample size was small, may contribute to future research.

Severe illness causing prolonged immobility and septic shock requiring steroids are associated with a high risk of developing muscle weakness (30). These conditions activate the ubiquitin-proteasome, calpain, and autophagy signaling pathways via systemic inflammatory cytokines. The resulting protein degradation overwhelms protein synthesis, leading to muscle wasting and inflammation-associated neuromuscular dysfunction (42). The detailed mechanism by which NMES prevents muscle wasting has not been elucidated; however, it is effective in preserving muscle size (43). NMES may increase mammalian target of rapamycin phosphorylation and decrease the messenger RNA expression of key genes involved in muscle protein breakdown (forkhead box protein O1) (44). NMES promotes muscle anabolism by accelerating the up-regulation of brain-derived neurotrophic factor and its tropomyosin receptor kinase B, and by enhancing the expression of cytoskeletal proteins, including tubulin and actin, as well as growth-associated protein 43 (45). Furthermore, NMES promotes nerve regeneration and axonal growth in response to nerve injury, and its combination with exercise has been reported to be effective in the early stages of nerve regeneration (46).

Our study has several limitations. It was conducted at a single-center in Japan and included only Japanese patients. Additionally, since the study focused on critically ill older adults, the effects of NMES may be limited in younger individuals with greater muscle reserves and shorter periods of immobility. Furthermore, differences in NMES device type, treatment duration, stimulation site, and timing of implementation could lead to varied outcomes (8, 9). The device used in this study induces widespread muscle contraction between the belts, enabling exercise of the entire lower limb skeletal muscle, and is characterized by being less painful compared with conventional NMES devices; it is commonly used in Japanese facilities (47–49). Therefore, it remains uncertain whether NMES would be acceptable to multinational patient populations, leaving room for discussion regarding the generalizability of our findings. This study was initiated during the COVID-19 pandemic. During the study period, our ICU was repeatedly designated as a COVID-19 unit, which restricted the admission of patients with general medical conditions. Consequently, although the study period was extended, the target sample size could not be achieved. Nonetheless, due to the small variability in muscle strength within the study population and the large effect size detected, the potential scientific gain from further data collection was deemed minimal. However, due to the small sample size, random error cannot be ruled out, and the results should therefore be interpreted with caution. Therefore, a larger, multicenter study is warranted to validate the efficacy of the intervention. Although outcome assessors were blinded to group allocation, blinding of the physical therapists, the multidisciplinary team involved in early mobilization in the ICU, and the patients was not feasible. Therefore, the possibility of unintended influences on the intervention cannot be ruled out. To mitigate this, we adhered strictly to the protocol and made every effort to ensure standardized early mobilization and enhanced post-ICU rehabilitation were provided to all patients. Finally, because ICU patients often develop severe illness acutely, it is difficult to measure their true pre-illness QIS (2, 12, 50). It also remains unclear how QIS changes in the long term after discharge, as follow-up was not conducted. Further research is required to evaluate long-term changes in QIS and clinical outcomes associated with NMES in the ICU.

## CONCLUSIONS

In older critically ill patients admitted to the ICU, the addition of NMES to early mobilization effectively prevented muscle weakness and significantly improved the 6MWD and ADL performance. These patients may represent potential target responders to NMES.

## ACKNOWLEDGMENTS

We acknowledge the study participants and the ICU and rehabilitation staff of the Kochi Health Sciences Center for their invaluable contributions to this study.

## Supplementary Material
