# Supplementary material for: Effect of Neuromuscular Electrical Stimulation for Older Critically Ill Patients in the ICU: A Randomized Controlled Trial
Source: Crit Care Explor. 2025 Nov 25;7(12):e1345. doi: 10.1097/CCE.0000000000001345 (PMC12657045; doi:10.1097/CCE.0000000000001345)
Supplement: Supplementary file 1 [file cc9-7-e1345-s001.pdf]

Supplemental Digital Content

Effect of Neuromuscular Electrical Stimulation for Older Critically Ill Patients in the Intensive Care Unit: A Randomized Controlled Trial

Authors : Kazuhiro Yokobatake, MS[1]; Hiroaki Kitaoka, MD, PhD[2]; Atsushi Morizane, MD, PhD[3]; Kensaku Kashima, MS[1]; Daichi Nishimori[1]; Shingo Nishimura[1]; Yumi Sakyo[4]; Shinya Takeuchi, MD, MPH[5]; Yasumasa Kawano, MD, PhD[6]; Tomoko Sugimura, MD, PhD[7]

[1]Department of Medical Technology Rehabilitation, Kochi Health Sciences Center, Kochi, Japan.
[2]Department of Cardiology and Geriatric, Kochi Medical School, Kochi Japan.
[3]Critical Care and Emergency Center, Kochi Health Sciences Center, Kochi, Japan.
[4]Department of Nursing, Kochi Health Sciences Center, Kochi, Japan.
[5]Department of Disaster and Emergency Medicine, Kochi Medical School, Kochi Japan.
[6]Department of Emergency and General Medicine, Fukuoka University Chikushi Hospital, Chikusino, Japan.
[7]Department of Emergency Medicine, Graduate School of Medicine, University of the Ryukyus, Okinawa, Japan.

Corresponding author and Email:
Kazuhiro Yokobatake
E-mail: yokoba.1008@gmail.com

Table of Contents: Supplemental Material	
Page Number	Content
Page 3-10	Study Protocol
Page 11	Supplementary Picture
Page 12	Open Science

study protocol

Effect of neuromuscular electrical stimulation for elderly critical ill patients in Intensive Care Unit：a randomized controlled trial

lead principal investigator: **Kazuhiro Yokobatake, MS**

Organization: Department of Medical Technology Rehabilitation,
Kochi Health Sciences Center, Kochi, Japan.

Background to the study

With recent advances in medicine, the number of patients surviving and being discharged from the ICU has increased. However, prolonged bed rest often leads to muscle weakness, generalized disuse syndrome, progression of post–ICU syndrome (PICS), ICU-acquired weakness (ICU-AW), and sarcopenia, which in turn result in long-term declines in activities of daily living (ADL) and quality of life (QOL)([1-4]).

Moreover, with the aging of the population, this issue has become an urgent concern, particularly among older patients. As a countermeasure, early rehabilitation has increasingly been recommended in the field of intensive care([5,6]).

However, in severe cases, patients are often required to remain at rest due to ongoing medical treatments, and early mobilization training is frequently delayed. While interventions such as nutritional therapy and bed-based exercise have been implemented, neuromuscular electrical stimulation (NMES) has recently gained attention for the prevention of muscle weakness, with numerous randomized controlled trials, meta-analyses, and systematic reviews being published ([7-9]). In the ICU setting, studies on NMES have yielded both positive and negative results, and a consistent consensus has yet to be established. The 'Expert Consensus on Early Rehabilitation in Intensive Care' in Japan ([6]) states that while adding electrical stimulation therapy to standard care may be effective in preventing muscle weakness compared to standard care alone, considerations regarding patient selection and treatment duration are necessary, and further evidence is required. Trethewey et al, ([7]) reported that the management and prevention of sarcopenia are crucial in the field of intensive care, and that effective interventions combining NMES and exercise may help preserve muscle mass and function in critically ill patients, emphasizing the need for high-quality RCTs.

RCTs investigating NMES interventions in the field of intensive care have often included relatively young patients with sufficient muscle mass reserves as study participants ([7–9]). Langeard et al. ([10]), in a systematic review of NMES intervention studies targeting older adults, reported that the muscle-strengthening effects of NMES are comparable to those of voluntary training. Kochi Prefecture is one of the most rapidly aging regions in Japan, and our emergency and critical care center ICU treats a large number of older and critically ill patients, making it feasible to conduct high-quality interventional studies in this population.

The objective of this study is to evaluate the effectiveness of early rehabilitation combining NMES with exercise therapy in critically ill elderly patients aged 65 years and older admitted to our emergency and critical care center ICU.

Study Design

single-center, randomized controlled trial

Randomization and masking methos

Block randomization will be employed. Random numbers will be generated by computer to create blocks of 2 or 4 participants for enrollment (n = 60; block size 2: 4 blocks, block size 4: 13 blocks). The allocation sequence will be prepared by personnel not involved in the treatment intervention. After patient enrollment, the intervention staff will open the allocation sequence to assign participants to groups. Participants will be randomly assigned in a 1:1 ratio to receive either NMES in addition to early mobilization (NMES group) or early mobilization alone (Control group). Although blinding of the NMES intervention will not be feasible, rehabilitation staff responsible for care after ICU discharge and outcome assessors will be blinded to group allocation.

Clinical research period

The study will be planned for 3 years from the start of patient enrollment.

Primary outcome

The primary outcome will be quadriceps isometric strength (QIS), normalized by body weight. QIS will be measured at two time points: when the patient is first able to get out of bed and at hospital discharge.

In the field of intensive care, the Medical Research Council sum scale (MRCss) is frequently used to assess muscle strength ([11,12]). However, it is a six-point ordinal scale that lacks quantitative objectivity, and issues such as a ceiling effect in grade 5 have been noted ([13,14]). For the assessment of lower limb muscle strength, evaluation using a hand-held dynamometer is considered to provide superior objectivity and reproducibility ([14–16]). It will also be regarded as effective for more detailed muscle strength assessment after ICU discharge and will be used as an outcome measure in this study.

Secondary Outcomes

・MRCss (when the patient is first able to get out of bed and at hospital discharge)

・Hand grip strength (when the patient is first able to get out of bed and at hospital discharge)

・A continuous walking distance at ICU discharge（no rest）

・6 minutes walking distance (at hospital discharge)

・Thigh circumference (15 cm above the knee) (at the initial intervention and hospital discharge)

・One-leg standing time (at hospital discharge)

・Barthel Index (at ICU discharge and hospital discharge)

・Clinical frailty scale (at hospital discharge and after six months)

Study population

<u>Inclusion criteria</u>

Patients will be screened upon ICU admission and will be enrolled within 24 hours if they:

(1) are ≥65 years old, and

(2) have an Acute Physiology Assessment and Chronic Health Evaluation II (APACHE II) score >20

<u>Exclusion criteria</u>

Patients will be excluded if they have:

(1) An expected ICU stay <72 hours

(2) A Clinical Frailty Scale (CFS) ≥ 5

(3) Inability to walk at discharge due to stroke, neuromuscular disease, or trauma

(4) Pacemaker implantation

(5) Poorly controlled malignancy

(6) A designated "Do Not Attempt Resuscitation" order

(7) Severe dementia

(8) COVID-19

(9) Suicidal attempt

(10) Inability to obtain consent

Sample size calculation

Among community-dwelling individuals over 75 years of age, the QIS is approximately 0.40 kgf/kg ([17,18]), whereas critically ill ICU survivors have been reported to have a QIS of 0.24 kgf/kg at discharge ([19]). Assuming a difference of 0.08 kgf/kg in QIS at discharge with a standard deviation of 0.1 kgf/kg, we calculated the required sample size with an alpha of 0.05 and a power of 80%. As a result, 26 patients per group were determined to be necessary, and we are aiming to enroll 60 patients to account for potential mortality and exclusions due to complications.

Intervention methods

NMES group; Early mobilization + NMES

Control group: Early mobilization only

Early mobilization program methods

Our ICU will adhere to the ABCDEF bundle ([19-21]) and will follow protocols based on a Japanese study on the safety of early mobilization ([22]). A five-step progressive program will begin with in-bed range-of-motion exercises and progress through sitting, standing, and walking. Early rehabilitation will be initiated within 48 hours of ICU admission and delivered by a multidisciplinary team comprising dedicated ICU physical therapists, board-certified intensivists (certified by the Japanese Society of Intensive Care Medicine), nurses, and clinical engineers. The team will hold daily conferences to review each patient’s status and set mobilization goals. If patients did not meet the discontinuation criteria and demonstrated the ability to walk, gait training was actively initiated. The early mobilization program will be implemented daily, and after ICU discharge, patients will receive enhanced rehabilitation provided by physical therapists and nurses to actively support the recovery of their ADLs. The early mobilization and rehabilitation sessions will last between 20 and 40 minutes, and exercise intensity will be progressively increased as long as the patient does not meet any discontinuation criteria. They will be provided seven days a week, every day. Once patients are able to transfer to a wheelchair or chair and do not meet any discontinuation criteria, resistance training using ankle weights will be actively implemented to strengthen the lower limb muscles. Exercise intensity will be adjusted as appropriate based on the 10-repetition maximum (10RM), and vital signs will be continuously monitored.

Early mobilization levels in our ICU

LEVEL 1 No mobilization or bed exercise

LEVEL 2 Sitting position in bed, including using a cycling ergometer and active range of motion

LEVEL 3 Sitting on the edge of the bed

LEVEL 4 Active transfer to the chair

LEVEL 5 Standing, stepping in place, or ambulating

Criteria for initiation of mobilization in our ICU

・Consciousness, $-3 \leq$ RASS $\leq +1$

・Respiratory rate, 5 breaths per minute $\leq$ RR $\leq$ 40 breaths per minute

・Percutaneous arterial oxygen saturation (SpO_2), $SpO_2 \geq 88\%$

・Fraction of inspiratory oxygen (F_IO_2), $F_IO_2 < 0.6$

・Positive end-expiratory pressure (PEEP), PEEP < 10 cmH_2O

・Not set for lung rest

・Heart rate, 40 bpm $\leq$ HR $\leq$ 130 bpm

・Mean arterial pressure. 60 mmHg $\leq$ MAP $\leq$ 100 mmHg

・Noradrenaline < 0.2γ, Dopamine < 8γ, Dobutamine < 8γ

・No recent new drug initiation or dose increase prior to the start

・No arrhythmia that could disrupt the hemodynamics

・No ECG changes suggestive of new myocardial ischemia or no untreated myocardial ischemia

・BT < 38.5ºC, Not undergoing hypothermia therapy

・No active bleeding, Hemoglobin concentration $\geq$ 7 g/dL

・Thromboembolism is under control

・No new-onset or uncontrolled organ ischemia

・Decision of intensivist or attending physician if No

Criteria for discontinuation of mobilization in our ICU

・Intolerable pain/ agony/ fatigue/Sudden dyspnea

・Reduced consciousness level compared to the start

・Distress, pallor of the face, and appearance of cyanosis

・RASS ≤ -3 or $+2 <$ RASS, Dangerous behaviors due to restlessness

・RR < 5 breaths per minute or RR > 40 breaths per minute

・SpO2 < 88%

・HR < 40 bpm or HR > 130 bpm

・MAP < 60 mmHg or MAP > 100 mmHg

・New arrhythmia requiring treatment, Suspected myocardial ischemia

・Suggested active bleeding

NMES protocol

Patients in the NMES group will receive NMES intervention in parallel with early mobilization, starting on Day 2 or within 48 hours of ICU admission.

NMES equipment:

Belt-type skeletal muscle electrical stimulation (G-TES®; Homer Ion Corp., Tokyo, Japan)

NMES settings:

A frequency of 20 Hz, a pulse width of 250 µs, and a duty cycle of five seconds of stimulation followed by a two-second pause will be used. The intensity will be set to the maximum level that induces visible muscle contraction without causing pain. NMES will be administered daily for 60 minutes. During treatment, a physical therapist, nurse, and intensivist will monitor the patient's responses, facial expressions, and vital signs. Efforts will be made to maximize muscle contraction during NMES. NMES intervention will be conducted daily until the patient is able to walk 100 meters continuously.

Criteria for discontinuation of NMES

・Intolerable pain/ agony

・Change in RASS

・Changes in vital signs

・Patients request

・If any symptoms are observed, the stimulation intensity will be appropriately adjusted

Contraindications and Prohibited Uses (as stated in the medical device package insert):

Do not apply the device to patients with implanted electronic devices (e.g., pacemakers) unless a medical opinion from a specialist is first obtained.

Do not apply to any other patients deemed inappropriate by the physician.

Expected Adverse Events

Discomfort, pain, redness, itching, or muscle injury caused by excessive electrical stimulation associated with the use of NMES.

Reporting of Adverse Events

If any of the above events are observed, the stimulation intensity will be gradually reduced or discontinued, and the patient will be monitored. Should an adverse event occur, it will be promptly reported to the attending physician and the department head, and appropriate measures will be taken. The event will also be reported to the ethics committee, which will deliberate on whether the study may continue.

Expected Benefits

The use of NMES may help prevent disuse muscle atrophy associated with prolonged bed rest, potentially leading to earlier independence in ADLs, improved physical function, and enhanced quality of life. NMES can be administered while the patient is in a supine position, and the procedure involves relatively simple tasks such as electrode placement, controller operation, monitoring for vital sign changes, and confirming muscle contractions. This simplicity may also reduce the burden on healthcare providers.

Expected Risks

Physical and psychological stress may occur due to discomfort, pain, redness, or itching associated with electrical stimulation.

Study Discontinuation

Individual Discontinuation Criteria

Participation in the study will be discontinued if：

・The participant withdraws consent

・A specific adverse event occurs

・The attending physician determines that continued participation is not appropriate

If discontinuation occurs, the participant will be monitored until their condition stabilizes.

Study-Wide Discontinuation Criteria

The entire study will be discontinued if：

・It is determined that continuation of the study is no longer beneficial

・A serious adverse event occurs that cannot be ruled out as unrelated and was not predictable

・The head of the research institution requests study termination

・A significant protocol violation is discovered

If the study is terminated, post-discontinuation monitoring will be conducted.

Conflict of Interest

This study is a collaborative project with Homer Ion Co., Ltd., which is providing the NMES equipment free of charge. The study will be conducted under a research agreement between Homer Ion Co., Ltd. and Kochi Health Sciences Center,, to be signed after obtaining approval from the ethics committee.

References

(1) Kizilarslanoglu MC, Kuyumcu ME, Yesil Y, et al. Sarcopenia in critically ill patients. J Anesth 2016; 30: 884–90.

(2) Zorowitz RD. ICU-Acquired Weakness: A Rehabilitation Perspective of Diagnosis, Treatment, and Functional Management. Chest 2016; 150: 966-971.

(3) Needham DM, Davidson J, Cohen H, et al. Improving long-term outcomes after discharge from intensive care unit: report from a stakeholders' conference. Crit Care Med 2012; 40: 502-9.

(4) Yende S, Austin S, Rhodes A, et al. Long-Term Quality of Life Among Survivors of Severe Sepsis: Analyses of Two International Trials. Crit Care Med 2016; 44: 1461-7.
(5) Devlin JW, Skrobik Y, Gelinas C, et al. Clinical Practice Guidelines for the Prevention and Management of Pain, Agitation/Sedation, Delirium, Immobility, and Sleep Disruption in Adult Patients in the ICU. Crit Care Med 2018; 46: e825-e873.
(6) Ad Hoc Committee for Early Rehabilitation, The Japanese Society of Intensive Care Medicine. Evidence based expert consensus for early rehabilitation in the intensive care unit. J Jpn Soc Intensive Care Med 2017; 24: 255-303.
(7) S.P. Trethewey, et al. Interventions for the management and prevention of sarcopenia in the critically ill: A systematic review. Journal of Critical Care 2019; 50: 287–295.
(8) Zayed Y, Kheiri B, Barbarawi M, et al. Effects of neuromuscular electrical stimulation in critically ill patients: A systematic review and meta-analysis of randomised controlled trials. Aust Crit Care 2019; 33: 203-210.
(9) Jones S, Man WD, Gao W, et al. Neuromuscular electrical stimulation for muscle weakness in adults with advanced disease. Cochrane Database Syst Rev 2016; 10: CD009419.
(10) Langeard A, Bigot L, Chastan N, et al. Does neuromuscular electrical stimulation training of the lower limb have functional effects on the elderly?: A systematic review. Exp Gerontol 2017;91:88-98.
(11) Fan E, Cheek F, Chlan L, et al: An official American Thoracic Society Clinical Practice guideline: the diagnosis of intensive care unit-acquired weakness in adults. Am J Respir Crit Care Med 2014; 190: 1437-46.
(12) Hermans G, Van den Berghe G: Clinical review: intensive care unit acquired weakness. Crit Care 2015; 19: 274.
(13) Baldwin CE, Paratz JD, Bersten AD. Muscle strength assessment in critically ill patients with handheld dynamometry: an investigation of reliability, minimal detectable change, and time to peak force generation. Journal of Critical Care 2013; 28: 77–86.
(14) Vanpee G, Hermans G, PhD, Segers J et al: Assessment of Limb Muscle Strength in Critically Ill Patients: A Systematic Review. Crit Care Med 2014; 42: 701-711.
(15) Roberson AR, Starkweather A, Grossman C, et al: Influence of muscle strength on early mobility in critically ill adult patients: Systematic literature review. Heart Lung 2018; 47: 1-9.
(16) Katoh M, Yamasaki H. Comparison of reliability of isometric leg muscle strength measurements made using a hand-held dynamometer with and without a restraining belt. J Phys Ther Sci, 2009,21: 37-42.
(17) Hayashida I, Tanimoto Y, Takahashi Y, et al: Correlation between muscle strength and muscle mass, and their association with walking speed, in community-dwelling elderly Japanese individuals. PLoS One 2014; 9:e111810.
(18) Chan OY, van Houwelingen AH, Gussekloo J, et al: Comparison of quadriceps strength and handgrip strength in their association with health outcomes in older adults in primary care. Age (Dordr) 2014; 36:9714.
(19) Marra A, Ely EW, Pandharipande PP, et al: The ABCDEF Bundle in Critical Care. Crit Care Clin 2017; 33:225-243.
(20) Lang JK, Paykel MS, Haines KJ, et al: Clinical Practice Guidelines for Early Mobilization in the ICU: A Systematic Review. Crit Care Med 2020; 48:e1121-e1128.
(21) Girard TD, Alhazzani W, Kress JP, et al: An Official American Thoracic Society/American College of Chest Physicians Clinical Practice Guideline: Liberation from Mechanical Ventilation in Critically Ill Adults. Rehabilitation Protocols, Ventilator Liberation Protocols, and Cuff Leak Tests. Am J Respir Crit Care Med 2017; 195:120-133.

(22) Liu K, Ogura T, Takahashi K, et al: The safety of a novel early mobilization protocol conducted by ICU physicians: a prospective observational study. J Intensive Care 2018; 6:10.

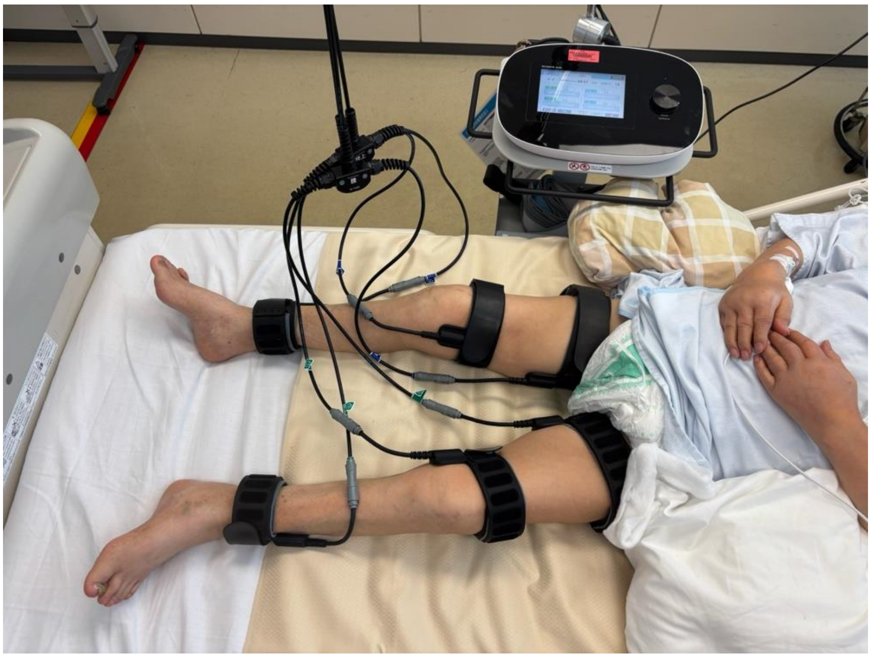

Supplementary Picture 1. Neuromuscular electrical stimulation. Belt electrodes were attached to the proximal and distal thighs and ankles.

Open science

Trial Registration Number

This study was registered in the University Hospital Medical Information Network Clinical Trials Registry (UMIN-CTR) prior to participant enrolment (registration no. UMIN000042154, registered on 19 October 2020).

Date sharing statement

All de-identified participant data will be made available to future researchers upon reasonable request to the corresponding author.
